# Optimized Methodology to Produce Platelet-Rich Plasma and Perform Platelet Aggregation in Patients With Coronary Artery Disease

**DOI:** 10.7759/cureus.69032

**Published:** 2024-09-09

**Authors:** Atul K Kashyap, Bonu Saikrishna, Bhanu Duggal

**Affiliations:** 1 Cardiology, All India Institute of Medical Sciences, Rishikesh, Rishikesh, IND

**Keywords:** clopidogrel, coronary artery disease, platelet aggregation, platelet-rich plasma, ticagrelor

## Abstract

Background: Inter-individual differences in clopidogrel metabolism and platelet counts within platelet-rich plasma (PRP) intrigued us to optimize light transmittance aggregometry (LTA) assay in coronary artery disease (CAD) patients administered with clopidogrel and ticagrelor. The objective of the study was to optimize PRP preparation using separating gel PRP tubes to perform LTA among CAD patients on clopidogrel and ticagrelor.

Methodology: Initially, we optimized PRP preparation and platelet aggregation (PA) on healthy controls. To validate the protocol, we recruited 10 healthy controls and 28 CAD patients, comprising 16 on clopidogrel and 12 on ticagrelor regimen. Bio-X, India, supplied PRP tubes (9 mL) with 3.2% sodium citrate. PRP and autologous platelet-poor plasma (PPP) were prepared by centrifugation at 151 g for seven minutes and 3,780 g for 10 minutes, respectively. LTA was performed using platelet aggregometer TA-4V (Stago, Asnières-sur-Seine, France). Adenosine diphosphate (ADP) (10 µM) was used as an agonist.

Results: The mean maximum platelet aggregation (MPA) among controls, clopidogrel patients, and ticagrelor patients were 55.161±13.69%, 53.17±22%, and 35.84±20.79% (p=0.042), respectively. The mean platelet volumes among groups were 9.34±2.51 fL, 7.60±0.85 fL, and 10.99±1.62 fL (p≤0.01), respectively.

Conclusion: We successfully optimized PRP preparation for the LTA assay using a PRP tube. Its application to measure PA routinely in a cardiology clinic seems propitious.

## Introduction

Antiplatelet drugs play a significant role in preventing vascular events. Clopidogrel is one of the most widely used antiplatelet drugs in combination with aspirin, but a limitation of clopidogrel is its variability in response across different ethnicities [1]. Clopidogrel is a prodrug that is metabolized in the liver by cytochrome P450 (*CYP450*) genes [2]. Its active metabolite binds irreversibly to the adenosine diphosphate (ADP) receptors on the surface of platelets and inhibits platelet aggregation and, thus, thrombus formation [3]. However, several mutations and single nucleotide polymorphisms have been reported in *CYP450* genes, which affect the adequate absorption of clopidogrel in the intestine. These modifications at the molecular level lead to heterogeneous responses to the clopidogrel regimen [4,5], causing variable platelet aggregation. The gold standard to measure platelet aggregation is light transmittance aggregometry (LTA), and the Platelet Physiology Subcommittee of the Scientific and Standardization Committee (SSC) of the International Society on Thrombosis and Haemostasis (ISTH) has provided certain recommendations for performing LTA. Nevertheless, the committee has not commented on the use of separating-gel platelet-rich plasma (PRP) tubes or monitoring antiplatelet therapy [6]. The preparation of PRP is strenuous, and the inter-individual difference in platelet count within PRP adds to the problem.

Recently, inconsistencies have been observed in the three PRP preparation kits, namely, Eclipse, Selphyl, and Emcyte, which are approved by the US Food and Drug Administration (US FDA). The authors observed that out of 51 blood samples, only one reached >50% platelet capture efficiency (PCE) [7]. The Emcyte kit showed a higher PRP concentration and a higher white blood cell count than others. Primarily, the authors reported variability in PRP products across different samples. In response to the abovementioned study, another group of researchers attempted to explain the inconsistencies by pointing toward the input sample volume, preparation method, and use of PRP-specific hematology analyzers [8].

Apart from the PRP preparation, another challenge is to optimize the LTA assay. High variability from different laboratories pertaining to the type of reagent, concentration, and pathological cutoff values necessitates the standardization of the LTA assay [9]. Despite existing standardization guidelines [6,10,11], surveys have indicated high inter-laboratory variability [12]. The scientific societies in the Netherlands adopted the SSC guidelines, intending to establish a national LTA protocol to detect platelet function disorders. A multicenter study conducted by these societies engaged 16 hospitals and recruited 120 healthy volunteers [13]. The study concluded that, at low agonist concentrations, LTA assay optimization does not necessarily lead to less variation. Therefore, LTA assay optimization remains challenging.

The present study aims to standardize PRP preparation by using gel-separating PRP tubes to perform an LTA assay. Routinely available 1.8 mL sodium citrate (3.2%) does not have a separating gel, whereas PRP tubes (10 mL) have a gel at the base and are prefilled with 3.2% sodium citrate. After centrifugation, three distinct layers of erythrocytes, gel, and PRP (bottom to top) can be observed. To the best of our knowledge, this is the first study to evaluate the application of gel-separated PRP tubes to perform an LTA assay.

## Materials and methods

This study was approved by the Institutional Ethical Committee of All India Institute of Medical Sciences (AIIMS), Rishikesh, Uttarakhand, India, and subjects were recruited from the Cardiology Inpatient Department (IPD). Patients diagnosed with coronary artery disease (CAD), post-percutaneous coronary intervention (PCI), and administered with either clopidogrel or ticagrelor were included. Subjects were recruited after obtaining informed consent. First, we standardized the PRP preparation and LTA assay on the blood samples of healthy individuals (not taking any antiplatelet drugs). Then, to further validate the modified protocol in a clinical setup, we recruited 28 patients with CAD who had been administered clopidogrel (n=16) and ticagrelor (n=12), and 10 healthy controls. LTA was performed within two hours of the collection of blood. All subjects were inhabitants of Uttarakhand or Uttar Pradesh (i.e., two states located in the northern part of India).

Reagents and apparatus

The following reagents and apparatus were used: 10 mL hypodermic syringe (B. Braun, Melsungen, Germany), PRP tube (Bio-X, Mumbai, India) (3.2% sodium citrate + separation gel), refrigerated centrifuge (Remi, Mumbai, India), 100 µL micropipette (Eppendorf, Hamburg, Germany), 1,000 µL micropipette (Eppendorf), micropipette tips, 1.5 mL microcentrifuge tubes, and 15 mL centrifuge tube (Corning, Corning, NY/Tarsons, Kolkata, India). To perform LTA, we used the platelet aggregometer TA-4V (Stago, Asnières-sur-Seine, France), ADP (Stago), glass cuvette and stirrer, and lint-free Kimwipes (Kimtech Science, Roswell, GA).

Preparation of the agonist

We used ADP as an agonist to perform the LTA assay; it is well known to induce primary and secondary platelet aggregation. ADP was supplied in a lyophilized form in a glass bottle and was hydrated using 1 mL double-distilled water (ddH2O) to produce a 20 µM concentration. ADP aliquots of 100 µL were prepared and stored at -20°C till further use. A working solution of ADP was prepared by adding 100 µL of 0.9% normal saline, and a final concentration of 10 µM was achieved.

Standardization of the PRP preparation

**Figure 1:**
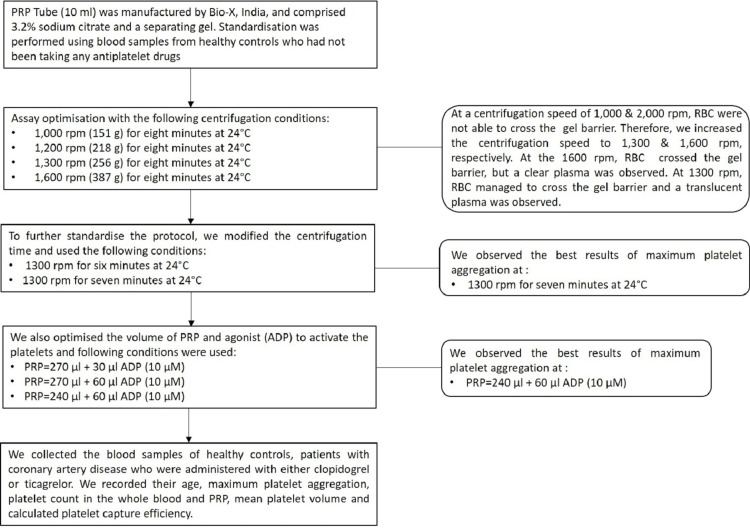
Flowchart depicting the optimization protocol PRP: platelet-rich plasma, RBC: red blood cell, ADP: adenosine diphosphate

We used different centrifugation conditions to standardize the PRP preparation for the LTA assay (Figure 1). We attempted centrifugation of blood on 151 g, 218 g, 256 g, and 387 g by keeping the time and temperature constant at eight minutes and 24°C, respectively. We observed the best results at 256 g, in terms of the separation of red blood cells (RBC) and plasma. To standardize the time variable, we centrifuged the blood samples at 256 g for six minutes and then seven minutes, keeping the temperature constant at 24°C. We observed a maximum aggregation of 71.77% in the sample spun at 256 g for seven minutes. However, an aggregation of only 35.42% was observed in the sample spun for six minutes. Platelet-poor plasma (PPP) preparation was performed by spinning the PRP at a high speed of 3,870 g since the objective was to remove platelets from the plasma. During the process of PRP preparation, we avoided agitating the samples as this might activate the platelets.

Modified LTA assay protocol

We slightly modified the LTA assay protocol to achieve maximum platelet aggregation (MPA) in the healthy control sample. According to the manufacturer's protocol, 30 µL of 10 µM ADP was added to the 270 µL of PRP. Although inconsistencies in platelet aggregation compelled us to improve the protocol further, we adjusted the volumes of PRP and ADP and measured the maximum platelet aggregation (MPA) at two different conditions: 270 µL (PRP) + 60 µL ADP and 240 µL (PRP) + 60 µL ADP (Figure 1). We kept the ADP concentration constant at 10 µM, and maximum aggregation was observed at 240 µL (PRP) + 60 µL ADP.

PRP and PPP preparation

A practicing nurse withdrew blood from the cubital vein/arterial sheath of the participants using a 10 mL syringe. The needle from the syringe was removed to avoid stress on platelets, and the collected blood was immediately transferred to a PRP tube containing 3.2% sodium citrate. The anticoagulant and the blood were slowly mixed by swirling the tube 4-5 times. The PRP tube was incubated for 30 minutes at room temperature, and the needle and syringe were carefully discarded. To obtain PRP, the PRP tube was centrifuged at 256 g for seven minutes at 24°C. The centrifugation was performed in a no-brake mode to avoid stress on the platelets. Erythrocytes and granulocytes were trapped under the separating gel upon centrifugation, whereas the PRP was observed as a supernatant. Approximately 2 mL of PRP was transferred to a 15 mL centrifuge tube, and around 900 µL of PRP was collected in a 1.5 mL tube. A total of 240 µL of PRP was carefully transferred to a glass cuvette containing a stirrer, and the rest of the PRP was used for platelet quantification. The cuvette was then placed onto the aggregation-measuring block that was pre-heated to 37°C. The PRP collected in a 15 mL tube was centrifuged at 3,780 g for 10 minutes at 24°C to prepare the PPP. After centrifugation, 300 µL of PPP was transferred to another glass cuvette and placed alongside the PRP tube on the measuring block.

LTA protocol

The LTA protocol was performed according to the manufacturer's guidelines with few modifications to the input ADP (10 µM) volume. ADP (20 µM) was thawed at room temperature for 30 minutes before being diluted with 100 µL of 0.9% normal saline. The diluted ADP (10 µM) was mixed thoroughly. PPP was measured first to set the baseline, and then, the PRP was measured. After placing the glass cuvette containing the PRP in the measuring block, we immediately added 60 µL of ADP (10 µM) and recorded platelet aggregation for six minutes. Schematics of this protocol are shown in Figure 1.

Statistical analysis

Continuous data were represented as mean±standard deviation (SD). Quantile-quantile (Q-Q) plots and the Shapiro-Wilk test were used to determine the distribution of the variables in the data. A comparison of the normally distributed variables was performed using a one-way analysis of variance (ANOVA), and multiple pairwise comparisons were carried out using a pairwise t-test. The non-normal distributed variables were compared using the Kruskal-Wallis test, and multiple pairwise comparisons were carried out using the pairwise Wilcoxon rank-sum test. Analysis was performed using the RStudio version 1.3.1093, and data were visualized using the ggplot2 package. p values less than 0.05 were considered significant.

## Results

**Figure 2:**
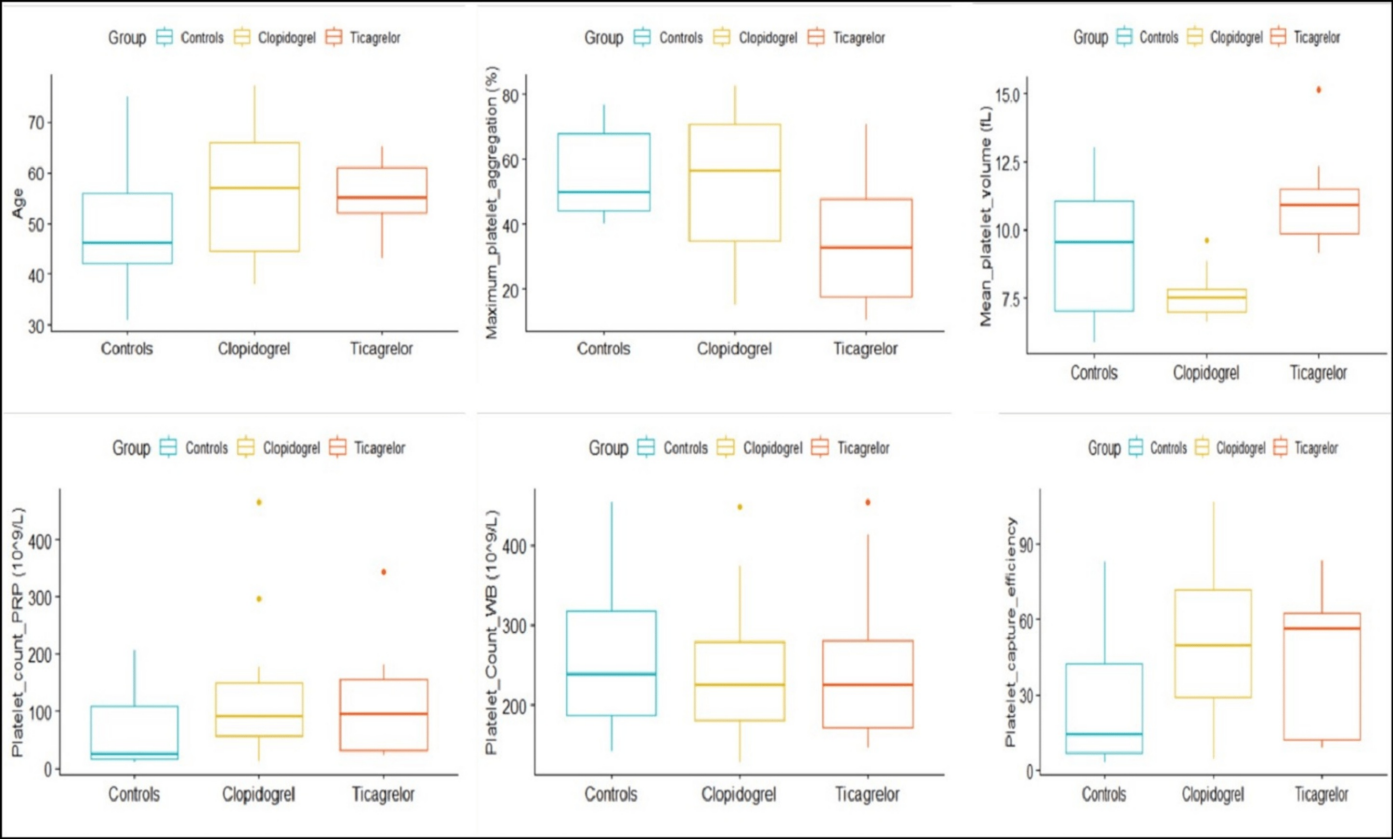
Box plots showing the distribution of variables across the three groups, namely, the controls, CAD patients on clopidogrel regimen, and CAD patients on ticagrelor regimen CAD: coronary artery disease

We standardized the PRP preparation method and adequately modified the LTA protocol to achieve maximum platelet aggregation (Table 1). This proposed method to prepare PRP is distinct from previously described protocols since we used specialized gel-separating PRP tubes. The best results were obtained under the following conditions: (1) for PRP preparation, centrifugation at 256 g for seven minutes at 24°C; (2) for PPP preparation, centrifugation at 3,780 g for 10 minutes at 24°C; and (3) for LTA protocol, 240 µL (PRP) + 60 µL ADP (10 µM). To further validate the protocol, we recruited 16 CAD patients administered with clopidogrel, 12 CAD patients administered with ticagrelor, and 10 healthy controls. The outcomes of the enrolled subjects are shown in Table 2, and the distribution of the variables is shown in Figure 2.

**Table 1:** Overall and pairwise comparison of variables among the groups *Statistically significant

Parameters	Group (mean±SD)			p value			
	Control	Clopidogrel	Ticagrelor	Control versus clopidogrel versus ticagrelor	Control versus clopidogrel	Control versus ticagrelor	Ticagrelor versus clopidogrel
Age	49.1±12.24	55.63±11.92	55.83±6.22	0.249	0.13	0.15	0.96
Maximum platelet aggregation (%)	55.161±13.69	53.17±22	35.84±20.79	0.042*	0.804	0.029*	0.028*
Mean platelet volume (fL)	9.34±2.51	7.60±0.85	10.99±1.62	<0.01*	0.014*	0.026*	<0.01*
Platelet count in PRP (Gl)	68.2±72.07	125.56±114.17	107.90±94.32	0.164	0.205	0.085	1.00
Platelet count in whole blood (Gl)	263.873±108.26	235.96±85.73	251.17±98.71	0.766	0.48	0.76	0.68
Platelet capture efficiency (%)	26.29±26.17	51.15±30.25	42.94±28	0.072	0.036*	0.08	0.507

**Table 2:** Platelet aggregation at varying PRP and ADP volumes among the controls, with centrifugation performed at 1,300 rpm for seven minutes at 24°C PRP: platelet-rich plasma, ADP: adenosine diphosphate, MPA: maximum platelet aggregation

Varying PRP and ADP (10 μmol) volumes	MPA (%)	Platelets in PRP
PRP - 270 µL + ADP - 30 µL	53.56	166,000
PRP - 270 µL + ADP - 60 µL	68.85	166,000
PRP - 240 µL + ADP - 60 µL	71.77	166,000
PRP - 270 µL + ADP - 30 µL	30.15	20,000
PRP - 270 µL + ADP - 60 µL	0	20,000
PRP - 240 µL + ADP - 60 µL	40.15	20,000
PRP - 270 µL + ADP - 30 µL	79.15	195,000
PRP - 270 µL + ADP - 60 µL	77.22	195,000
PRP - 240 µL + ADP - 60 µL	85.81	195,000

The mean ± SD of MPA (%) was 53.17±22 in patients treated with clopidogrel, and the value was 35.84±20.79 in patients treated with ticagrelor. The mean±SD of MPA (%) in the control group was 55.161±13.69. Significant differences were observed in the MPA among the three groups (p=0.042), whereas multiple pairwise comparisons between the control group and the clopidogrel group showed no significant differences (p=0.804). We also observed significant differences in the mean platelet volume among these groups (p<0.01). No significant differences were observed in the PCE between the clopidogrel group and the ticagrelor groups (p=0.507). However, significant p values were observed compared to the control and clopidogrel groups (p=0.036), as shown in Table 1. Non-significant differences were observed among the variables, such as age and the platelet count in PRP and whole blood.

## Discussion

Unequivocally, the literature suggests that optimizing LTA assay is challenging, and inconsistencies in the preparation of PRP add to the problem. To the best of our knowledge, gel-separating PRP tubes have not been coupled with LTA assay before, and this is the first report to optimize LTA assay using PRP prepared from gel-separating PRP tubes. The Platelet Physiology Subcommittee of SSC/ISTH published guidelines in 2013 pertaining to LTA assay. These guidelines discussed various aspects, including pre-analytical variables, preparation of PRP, PPP, and quality of PRP. The SSC/ISTH guidelines distinctively stated that the guidelines apply only to diagnosing bleeding disorders and that the use of LTA assay to monitor antiplatelet drugs would need further standardization [6]. We identified the lacuna and attempted to standardize the assay to measure platelet aggregation in a clinical setting.

Eclipse, Selphyl, and Emcyte are three US FDA-approved PRP preparation kits [7], and PRP is being used in dermatology clinics to treat alopecia [14]. The three aforementioned systems had a minimum-maximum PCE of 1.4%-52.8% (Eclipse), 8.1%-19.4% (Selphyl), and 13.5%-68% (Emcyte), and the input blood volumes were 22 mL, 27 mL, and 50-120 mL, respectively. We used the Bio-X PRP tube and observed a minimum-maximum PCE among the controls, the group administered with clopidogrel, and the group administered with ticagrelor of 3.27%-82.59%, 4.55%-106.45%, and 9.03%-83.05%, respectively. The input blood volume was 9 mL. Our results with low blood input are comparable to those of the three US FDA-approved systems. In line with Inyang et al. [7], we observed similar inconsistencies in the Bio-X PRP tube pertaining to PCE. The PCE minimum-maximum values were widely spaced, and the coefficients of variation among the group administered with clopidogrel, the group administered with ticagrelor, and the control group were observed as 0.59, 0.65, and 1, respectively.

The prevalence of clopidogrel resistance is reported to range from 5% to 40%. However, it is estimated as 70% in some of the Asian communities [15,16]. Our results with regard to the comparison of maximum platelet aggregation between the control and clopidogrel groups did not exhibit significant differences, which might be due to the high prevalence of clopidogrel resistance. Previous studies have reported that the frequency of the detrimental allele CYP2C19*2 was observed as 40.2 in the South Indian population [16,17]. Therefore, it would be interesting to investigate clopidogrel resistance using a combinatorial approach where platelet aggregation, CYP2C19-associated gene polymorphism, and plasma levels of clopidogrel active metabolite can be measured simultaneously in future research.

The limitation of the present study was that we could not perform comparative experiments between our proposed method and other platelet aggregation measuring systems. With respect to the PRP preparation, we could not compare the PCE of PRP tubes with other centrifugation methods. Although initially, we used routinely available 1.8 mL sodium citrate (3.2%) tubes, inconsistencies were observed when measuring the MPA. A multicenter study to monitor antiplatelet drug therapy utilizing LTA assay and other techniques in a clinical setup is lacking. A large study can certainly further establish the population-specific cutoff value and help guide antiplatelet therapy. This may reduce post-percutaneous coronary intervention (PCI) complications and mortality. Another possible limitation of this study could be the lack of data regarding the social determinants of health (SDH), such as income and social protection, education, and unemployment. Since CAD is a lifestyle disorder, SDH data could have shed light on the risk factors, affordability, and access to medical services. Our standardized method, along with other platelet aggregation assays and genomic studies, can aid in developing population-specific reference values.

## Conclusions

In conclusion, this study successfully optimized the preparation of PRP using separating gel PRP tubes for the LTA assay in patients with CAD who are administered with clopidogrel and ticagrelor. The study found significant differences in platelet aggregation and platelet volumes among healthy controls, and clopidogrel and ticagrelor patients. The optimized LTA assay using PRP tubes can be used routinely in a cardiology clinic to measure platelet aggregation, which can aid in the management of CAD patients.
